# Health hazards of China’s lead-acid battery industry: a review of its market drivers, production processes, and health impacts

**DOI:** 10.1186/1476-069X-12-61

**Published:** 2013-08-03

**Authors:** Tsering Jan van der Kuijp, Lei Huang, Christopher R Cherry

**Affiliations:** 1 State Key Laboratory of Pollution Control and Resource Reuse, School of the Environment, Nanjing University, Xianlin Campus, Nanjing 210023, China; 2 Associate Professor of Civil and Environmental Engineering, University of Tennessee-Knoxville, Knoxville, Tennessee 37996-2010, USA

**Keywords:** Lead-acid battery, China, Pb, Lead pollution, Lead poisoning, Heavy metals

## Abstract

Despite China’s leaded gasoline phase out in 2000, the continued high rates of lead poisoning found in children’s blood lead levels reflect the need for identifying and controlling other sources of lead pollution. From 2001 to 2007, 24% of children in China studied (N = 94,778) were lead poisoned with levels exceeding 100 μg/L. These levels stand well above the global average of 16%. These trends reveal that China still faces significant public health challenges, with millions of children currently at risk of lead poisoning. The unprecedented growth of China’s lead-acid battery industry from the electric bike, automotive, and photovoltaic industries may explain these persistently high levels, as China remains the world’s leading producer, refiner, and consumer of both lead and lead-acid batteries.

This review assesses the role of China’s rising lead-acid battery industry on lead pollution and exposure. It starts with a synthesis of biological mechanisms of lead exposure followed by an analysis of the key technologies driving the rapid growth of this industry. It then details the four main stages of lead battery production, explaining how each stage results in significant lead loss and pollution. A province-level accounting of each of these industrial operations is also included. Next, reviews of the literature describe how this industry may have contributed to mass lead poisonings throughout China. Finally, the paper closes with a discussion of new policies that address the lead-acid battery industry and identifies policy frameworks to mitigate exposure.

This paper is the first to integrate the market factors, production processes, and health impacts of China’s growing lead-acid battery industry to illustrate its vast public health consequences. The implications of this review are two-fold: it validates calls for a nationwide assessment of lead exposure pathways and levels in China as well as for a more comprehensive investigation into the health impacts of the lead-acid battery industry. The continuous growth of this industry signals the urgent need for effective regulatory action to protect the health and lives of China’s future generations.

## Background

Despite its well-documented health impacts and efforts to curb its use, lead (Pb) remains a pervasive global neurotoxin capable of causing serious and in some cases irreversible neurological damage. For years, leaded gasoline was the dominant source of human exposure to Pb: it had accounted for 80-90% of airborne Pb in cities where it was used [1]. Since a global phase out of leaded fuels began in the mid-1970s, blood lead levels (BLLs) have plummeted worldwide. In the United States, for example, the prevalence of elevated BLLs (at the time, ≥100 μg/L, 100 micrograms of Pb for each liter of blood) decreased from 77.8% to 4.4% between 1976–1980 and 1988–1991 [2]. Still, 16% of children worldwide are currently estimated to have BLLs above 100 μg/L [3]. Although leaded gasoline in China was phased out on July 1, 2000, approximately 24% of China’s children from 2001–2007 were assessed to have elevated BLLs [4], a noticeable drop from a previous 1995–2003 assessment showing 34% with elevated levels yet still unacceptably high by global averages.

Determining the sources of these high exposure levels will have critical implications for future public health and risk reduction measures. The rapid development of China’s lead-acid battery (LAB) industry may be responsible for these persistently high BLLs. China’s LAB industry is the world’s largest in terms of production and consumption, occupying over 30% of global LAB output [5,6] and using over 67% of China’s total Pb production [7]. Despite these trends and the well-documented Pb problem in China, the current literature lacks aggregate data and assessments of the health hazards of China’s LAB industry. It also does not evaluate the LAB industry’s potential links to numerous Pb poisoning outbreaks. Moreover, no existing body of research has taken the multi-angled approach of integrating the market factors, production processes, and health impacts of the LAB industry. As a result, this paper is the first to integrate these system components to demonstrate the public health implications of this growing industry.

This review assesses the role of China’s rising LAB industry on Pb pollution and exposure. It begins with a synthesis of biological mechanisms of Pb exposure followed by a discussion of the three main new market drivers of the LAB industry’s growth in China: the electric bike market, the automobile market, and photovoltaic systems. It then categorizes the four main industrial processes responsible for Pb pollution from the LAB industry and presents the geographic (province-level) distribution of LAB production activities. Next, a brief literature review reveals potential links between recent mass Pb poisonings and the LAB industry. The paper closes with a discussion of China’s new policies to address the LAB industry and identifies policy frameworks that can mitigate exposure to Pb.

### Biological mechanisms and health impacts

The United Nations Environment Programme labels Pb a “potent neurotoxin” and a “nerve poison” that globally threatens the health and intellectual development of millions of children and adults [8]. It is a potentially lethal neurotoxin that affects virtually every organ in the human body, crossing the blood–brain barrier by mimicking calcium ions in order to access the central nervous system. Here, it can inflict brain damage, mental retardation, nervous system disorders, encephalopathy (abnormal brain function), cell function deterioration, and a host of other neurological disorders [9,10]. Until the toxin is eradicated, it will continue to impair sensory and cognitive functions [11,12], cause serious kidney and cardiovascular damage [7], and disrupt overall organ development, particularly in children [13]. Gastrointestinal, cardiovascular, reproductive, and nervous systems can all be impacted even if only small doses infiltrate the body [14]. The cumulative and degenerative characteristics of Pb can generate adverse health effects in every individual, particularly in children, who are most susceptible to its long-term effects.

Several physiological and behavioral factors are responsible for this exposure susceptibility. First, the underdeveloped nervous systems of children have not yet acquired the detoxification capabilities to offset the effects of Pb. In adults, approximately 99% of absorbed Pb will be excreted naturally within a couple weeks, but in children, only about 30% of absorbed Pb will be eliminated in waste [15]. Under conditions of continued exposure, most of the original Pb will be retained and more will continue to accumulate in the body tissues of children. Second, in addition to weaker Pb detoxification capabilities, children have higher Pb absorption rates than adults, meaning that the more a child is exposed to Pb, the more readily it enters and remains in a child’s body. The oral absorption rate of Pb in the gastrointestinal tract for children ranges between 40% and 50%, whereas adults absorb between 10-15% [8,16]. Moreover, a greater proportion of circulating Pb gains access to the brains of children, especially those under 5 years of age [17]. Third, since children have higher inhalation rates per unit of body mass than adults [18], they remain disproportionately vulnerable to Pb exposure via inhalation pathways. Finally, some behavioral characteristics of young children such as crawling and frequent hand-to-mouth activity will increase rates of exposure to Pb, including contaminated soils and paints.

Numerous studies have indicated that there is no safe threshold for exposure to Pb, i.e., there is no amount too small to induce an adverse biological reaction [19,20]. Based on these findings, as of May 2012 the U.S. Centers for Disease Control and Prevention lowered the reference value for identifying exposed children from 100 μg/L to 50 μg/L [21]. Because of children’s unique susceptibility to Pb absorption, even relatively low levels of blood concentration (≤100 μg/L) can lead to permanent intellectual impairment and organ system failure [22]. Therefore, regulators must identify the primary sources of Pb pollution and work to reduce the risk of Pb exposure in children.

### Market drivers of China’s LAB industry

Globally, Pb derives either from primary (mining) or secondary sources (recycling and refining). The vast majority of Pb (~80%) in global commerce is used to produce LABs, and 97% of these batteries are recycled and reprocessed, primarily in developing low-income countries [3]. China is the world’s largest producer, refiner, and consumer of Pb, with over 67% (>1.92 billion kg) of its total Pb usage allocated towards producing LABs [7]. Some estimates reach as high as 70-75% [23,24]. The remaining uses of Pb in China go towards producing glass, household items, lead alloys, cables, paint additives, and anti-corrosion materials.

**Figure 1:**
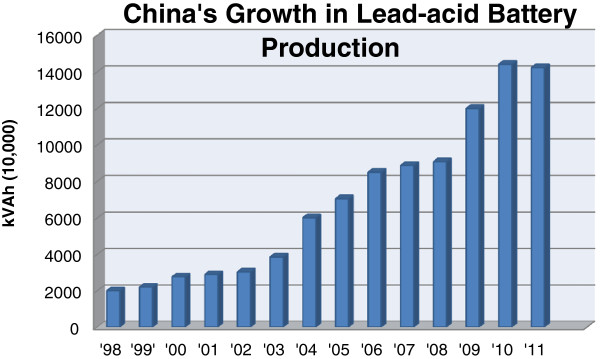
**China’s yearly growth in lead-acid battery production by kilo-Volt Amp hours.** Generated from industrial data collected by *China Metal Bulletin*.

Due to their relatively low-costs and high power surge capabilities, LABs are used to fuel growing demand for electric bikes (e-bikes) and motor vehicles. Other applications include powering photovoltaic (PV) devices, uninterruptible power supplies, telecommunications technologies, and electric power systems. With the rapid development of each of these industries in China, the production of LABs is poised to increase dramatically, as is the concomitant rise in Pb pollution. Figure 1 depicts this steady growth in China’s LAB production industry from 1998–2011 [25].

**Figure 2:**
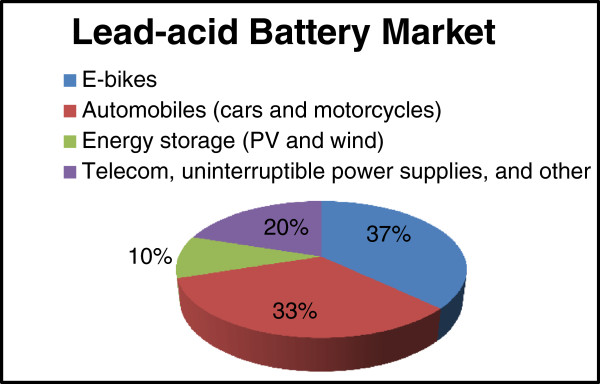
**The market distribution of lead-acid batteries in China in 2011.** Adapted from data and statistics compiled by Occupational Knowledge International.

Although price fluctuations and environmental regulations alter supply and demand flows, the LAB industry in China shows no signs of decline despite the availability of competing alternatives such as lithium-ion batteries. The China Environment Forum reported that two factors are primarily responsible for the LAB industry’s annual growth rate of 30% in China [26,27]:Figure 2 displays estimates of China’s LAB market distribution for the year 2011 [24].

1. Increased global demand for automobiles.

2. Increased utility in domestic technologies such as e-bikes and PV systems.

### Electric bike market

Fueled by consumer demand for inexpensive and convenient transportation, China’s e-bike market is the largest in the world. The relatively lower cost of electricity compared with gasoline, as well as the convenience of recharging e-bike batteries with a standard electrical outlet, make e-bikes one of China’s fastest growing modes of transportation. Moreover, many local governments are promoting the use of e-bikes in urban centers due to their zero tail-pipe emissions and ability to cut down on traffic congestion. These factors have contributed to well over 100 million e-bikes purchased and on the road in the past decade [28]. Currently, China is producing approximately 30 million e-bikes per year. Energy consulting firm Pike Research estimates that by 2018, annual e-bike sales in China will surpass 42 million, giving the nation almost 90% of total world market share [29].

Over 95% of e-bikes sold in China use rechargeable LAB technology [30]. E-bike batteries are about the same size as a typical car battery, containing 10.3-14.7 kg of Pb that comprises 70% of the battery’s total weight and requires replacement every one to two years, or up to 10,000km [31]. Thus, due to the shorter lifespan and high replacement rate of e-bike batteries, lifecycle Pb loss rates per kilometer for e-bikes significantly exceed those for other vehicles.

### Automobile market

As the world’s largest producer and consumer of Pb and automobiles, China’s automotive market continues to expand rapidly. In 2010 alone, China produced over 18.2 million passenger cars, doubling the second largest producer Japan [32]. Production trends will increase greatly over the next few decades to keep pace with China’s expanding vehicle market. By the end of 2011, car ownership in China exceeded 100 million [33] and under simulated growth models, China’s vehicle population would reach 185, 364, and 607 million by 2020, 2030, and 2050, respectively [34]. Long-term data projections from a separate study reveal similar results: 321 million to 391 million cars in China by 2035, and 486 million to 662 million by 2050 [35].

With these stark projections in both car production and consumption, there will be a concomitant increase in LAB production to power these vehicles. Each car battery contains 14 kg of Pb and has components similar to e-bike batteries. However, automobile batteries have a 5× greater lifespan, resulting in less frequent recycling rates and ultimate Pb loss.

### Photovoltaic (PV) systems

Under China’s landmark 2005 Renewable Energy Law, the proportion of energy generated by renewables is expected to increase dramatically over the next two decades. To meet these ambitious targets, China’s National People’s Congress has established 5 GW as an official minimum PV target for 2015, with a longer-term target of 20 to 30 GW by 2020 [36].

By the end of 2011, China had already installed a cumulative PV capacity of 3.3 GW, representing a more than 400% increase from the prior year [37]. PV systems require energy storage systems (batteries) to improve reliability and avoid fluctuations in power supply. 75% of these PV systems deploy lead-acid batteries, causing an expansion in LAB production and toxic Pb emissions [38]. Rural home-based systems and decentralized power grids in western China will rely on PV systems as a cost-effective power supply. This heavy reliance on LABs is not expected to change in the coming years, as the costs of the primary alternative lithium ion battery currently remain two to four times higher per kWh than LABs [39].

Given that China’s solar installations require 55 kg of battery weight (36 kg of Pb) per kW-year of installed PV capacity [40], the government’s plan to nearly quadruple current capacity in the coming decade reflects how rapidly both the PV and LAB markets are growing. Thus, it is critical to examine how China’s far-reaching targets for PV expansion will impact the LAB market.

### Industrial production and pollution processes

Pb can be emitted into the environment during four main production stages: mining/concentrating, smelting/refining, manufacturing, and recycling (secondary refining). In a review accounting for global losses of anthropogenic Pb, China was found to be the leader of Pb emissions in Asia. Furthermore, China ranks highest globally in the first and second production stages as well as in overall Pb waste emissions [41]. A study analyzing specific Pb loss rates during the LAB production process attributed 52.0% of total losses to mining/concentrating, 19.5% to primary refining, 15.0% to manufacturing, and 13.5% to secondary refining [42]. These Pb loss rates account for the Pb that falls outside the LAB production cycle and enters the environment, frequently without prior adequate treatment.

In China, loss rates along the entire industrial supply chain result in more than 30% of the Pb content of a battery lost to the environment [42]. Meanwhile, the organized recovery of used LABs is less than 30% [23], meaning that the remaining 70% is either discarded into the environment or collected and processed by informal, unregulated channels. Due to recycling inefficiencies, the latter often results in loss rates of up to 50% of the battery’s Pb content [43]. Understanding these loss rates is crucial for locating and targeting the most harmful production processes that pose the biggest risks to human health.

**Figure 3:**
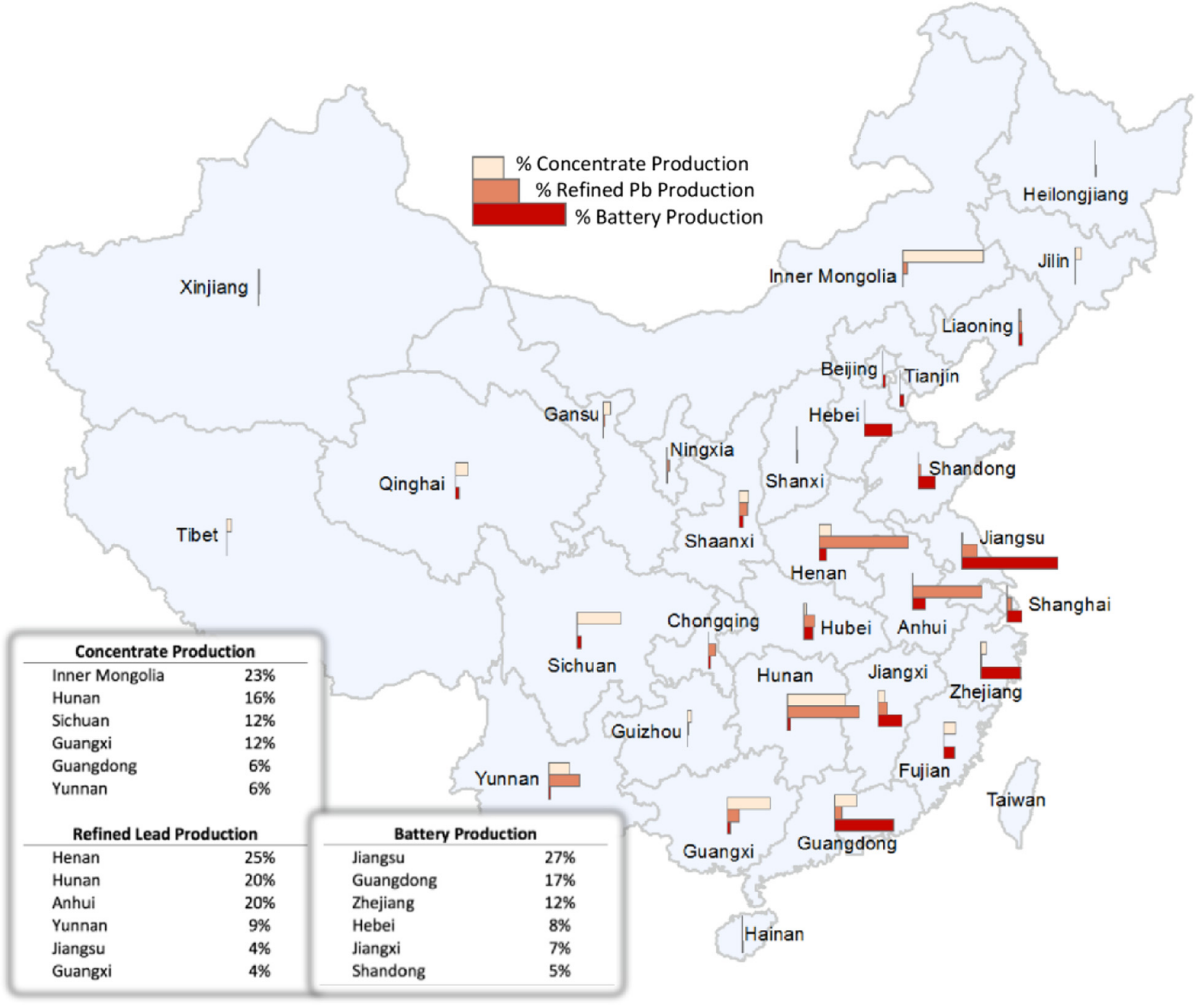
**Geographic distribution of China’s concentration, refining, and battery production activities.** The top six provinces in each production process are listed, accounting for >75% of production in each process. Most mining, concentrating, and refining processes occur in China’s interior. Battery manufacturing tends to occur along the coast. Figure is produced from raw industrial data obtained from China Statistical Yearbooks and provincial Environmental Protection Bureau statistics.

Figure 3 shows the geographic distribution of China’s Pb concentrating, refining, and battery manufacturing activities in 2010, derived from data presented in [44,45].

### Lead mining and concentrating

Most Pb mining deposits are located in Hunan, Yunnan, Sichuan, Guangdong, Guangxi, Henan, and Anhui provinces as well as key areas in northeast and northwest China [5]. In 2010, over 75% of China’s Pb concentrate output derived from six provinces: Inner Mongolia, Hunan, Guangdong, Sichuan, Guangxi, and Yunnan. Total Pb concentrate production in 2010 was 1.7 billion kg, a 13% increase from the 1.5 billion kg produced in 2008 [46].

The main three lead-bearing minerals galena (lead sulfide), cerrusite (lead carbonate), and anglesite (lead sulfate) are extracted from underground mines. Once the ore is removed, it is treated at a concentrating mill, which removes the waste rock (tailings) from the Pb by crushing the ore into granular pieces. After this process, tailings are either discharged into holding ponds resembling artificial albeit toxic lakes or discarded into the environment. Although most of the Pb is already extracted, tailings still contain residues of Pb and other heavy metals.

The finely crushed ore is diluted with water to form a slurry mixture and then poured into a flotation cell, into which chemicals (e.g. xanthate) are added to separate the waste rock from the metals. The waste rock, or gangue, sinks to the bottom of the flotation cell while the metal particles float to the surface and create Pb concentrate for refining. The gangue is discharged into holding ponds similar to those that collect tailings. This step in the production process produces the most Pb loss, measured by inefficiencies in extracting Pb concentrate from ore. To the extent that holding ponds are well-contained and well-engineered, the impacts of the mining process can be localized to mine workers and surrounding communities. However, poorly-managed tailing and gangue disposal processes can contaminate water and soil and transport pollution to larger populations.

### Lead smelting/refining

The six provinces that accounted for over 81% of total refined Pb output in 2010 are Henan, Hunan, Yunnan, Anhui, Guangxi, and Jiangsu provinces. Total refined Pb production in 2010 was 3.8 billion kg, a 19% increase from the 3.2 billion kg produced in 2008 [46].

During this stage, all lead-bearing elements along with sand and limestone are unloaded onto a sinter plant, where super-heated air (about 1,400°C) “roasts” the concentrate, fusing it into brittle material called sinter. Once sufficiently refined, the Pb compound is cooled and alloyed with antimony to increase strength and durability.

During the refining/roasting process, Pb particles not captured in the sinter are released into the air. Unless baghouse filters are installed to collect these pollutants, toxic emissions will result in air pollution. A solid waste byproduct of this Pb purifying process, slag is a dense substance that is more toxic than gangue and must be secured and monitored closely to avoid human exposure. The substance is often discarded into landfills or the environment, as it serves little industrial purpose.

### Lead battery manufacturing: oxide and grid processing, plate processing, and battery assembly

Based on total economic output and battery production capacity for 2010, about 75% of LAB production is supplied by six provinces: Jiangsu, Zhejiang, Guangdong, Hebei, Jiangxi, and Shandong. Zhejiang and Guangdong were also sites of recent mass Pb poisonings of children, resulting in the closure of over 300 battery manufacturers in May 2011. Specific regional hubs of LAB manufacturing are concentrated in the Changxing region of Zhejiang, the Baoding region of Hebei, the Pearl River delta area of Guangdong, the Quanzhou region of Fujian, the Jiyuan region of Henan, the Subei region of Jiangsu, and the Jiaodong region of Shandong [5]. Total consumption of Pb in China has increased by an average of 20% per year from 1999 to 2009 and is estimated to be about 3.86 billion kg in 2009 [46].

Flooded LAB manufacturing first requires making lead oxide paste from the refined Pb by adding water, acid, and a host of other chemicals. Meanwhile, grids are cast from lead alloy and molten lead, which are then combined with the paste. This process prepares lead plates for assembly into batteries. The final procedures involve hydrosetting, enveloping the plates with porous membranes, and assembling the final product.

A LAB contains an anode and cathode made from Pb, components that are bridged partially by a solution of sulfuric acid. When energy is produced, chemical reactions create toxic lead sulfate. Pb is the primary component (by weight), constituting 60-70% of the battery’s mass.

Although Pb loss rates due to manufacturing remain lower than the mining and smelting stages, locations near population centers can cause high levels of Pb exposure. LAB manufacturing enterprises often take advantage of lax regulatory enforcement by moving into poorer, rural communities. Consequently, local residents are directly exposed to the Pb exhaust produced during the casting process that escapes into the atmosphere. Another major source of Pb exposure is from lead oxide that escapes from the paste mixing machine, which dries and becomes airborne. In all major stages of manufacturing, Pb exhaust may be released as air pollution or waste mixtures may be discharged into soil and waterways.

### Lead battery recycling

The top manufacturers of secondary LABs are located in Henan, Jiangsu, and Hubei provinces [5]. Pb that is not discarded as waste into landfills or the environment is often recycled by unregulated small-scale recycling enterprises that can operate almost anywhere. Therefore, assessing the geographic distribution of secondary LAB production remains exceedingly difficult. In 2009, total secondary Pb production accounted for 35% of total refined Pb production (1.3 billion kg) in China, compared with 22% in 2005, and is expected to approach 50% of total refined Pb output by 2015 [46]. Consequently, most Pb will still derive from more inefficient and polluting primary production processes. Despite the cost-effectiveness of recycling Pb, the process is not without its environmental hazards.

LAB recycling is a relatively simple process. After the battery is crushed into small pieces and washed, Pb compounds are manually or mechanically detached from the plastic and sulfuric acid solution. The Pb is melted and then sent to LAB manufacturing facilities for reprocessing, while the acid is neutralized by an alkaline solution or diluted with water.

In addition to the emissions produced by remanufacturing and reprocessing, LAB recycling creates Pb fumes when the spent battery is heated and melted. These fugitive emissions then escape into the atmosphere and eventually settle on soil, which results in widespread persistent contamination. The wastewater produced during the battery washing process can contain a host of heavy metals. At large-scale enterprises, this substance may be sent to wastewater treatment facilities; however, most small-scale recyclers, accounting for over 50% of total LAB recovery, often discharge the toxic wastewater directly into the environment, contaminating local water sources [5].

### Links between lead poisoning and the LAB industry

China faces a public health and social stability challenge with regard to Pb poisoning, especially in children. Scores of mass Pb poisoning incidents involving children have directly fueled large-scale protests, resulting in factory damage and violent riots. A comprehensive review of children’s blood lead levels in China demonstrated that from 1995 to 2003, the mean BLL of children was 92.9 μg/L, and that 34% of the subjects had BLLs higher than 100 μg/L. This level stands multiple times higher than the mean found in developed countries (30 μg/L) [47]. A similar review conducted by the same authors found that from 2001 to 2007, the mean BLL of the children studied (N = 94,778) was 80.7 μg/L, and that 24% (24,065 children) had levels exceeding 100 μg/L [4]. China’s child population (<14 years old) is 260 million [48]. If almost a quarter of those children (65 million) are overexposed to Pb, then millions could face the risk of lifelong developmental and neurological disorders.

The high rates of Pb poisoning found in children’s BLLs continue to reflect the need for identifying and controlling other sources of Pb pollution besides leaded gasoline. From 2009 to 2011 high-profile Pb poisoning incidents in several provinces of China highlighted the impacts on more than 4,000 children, with numerous studies documenting high BLLs in working populations within Pb smelter and mining areas as well as LAB factories [7]. Furthermore, Chinese children living in industrial zones have significantly higher average BLLs than those in urban and suburban areas. The Pb in these industrial areas largely originates from Pb smelting, Pb battery production, and other Pb-related industrial operations [4].

A major review of Pb pollution and poisoning cases integrated Pb exposure data from 618 papers (1990–2005), covering 23 of the 31 administrative regions of China [49]. The review focused on LAB factories and Pb smelters, underscoring the prominent role the LAB industry plays in Pb exposure. The vast majority of the studies reported from the industrial areas of coastal provinces in eastern China: Fujian, Zhejiang, Jiangsu, Shandong, Guangdong, and Shanghai municipality. This geographical concentration was primarily due to the large number of Pb-producing facilities in these areas. The study concluded that from 2003–2005, the Pb poisoning rate for occupationally-exposed LAB workers in China was 36.8%, down from an already elevated rate of 45.0% from 1990–2002.

Another study conducted in May 2011 centered on a large LAB manufacturing factory in Heyuan, Guangdong Province. Several hundred residents had BLLs exceeding 100 μg/L due to exposure to the facility’s waste discharges, suggesting that LAB production at this factory prompted these elevated BLLs to occur among the neighboring populations, rather than just the working population [7]. The study further concluded that BLLs in the clean reference area (1000–2500 meter distance from factory) were significantly lower than those found in the polluted area (0–500 meter distance from factory) and second, that BLLs declined with increasing distance from the factory.

Dozens of studies have linked industrial sources of Pb pollution to Pb’s associated health impacts, including those caused by the LAB industry. A global assessment reveals a marked presence of small-scale LAB recycling operations in the developing world. Surveys in Jamaica, the Philippines, and the Dominican Republic show clear associations between children who live near or even work in LAB recycling facilities and significant elevations in BLLs- levels almost five times higher than those in unexposed children [50]. Unlike China, which participates in all four stages of LAB production, most developing countries rely on recycling exports of used LABs to sell back to the global Pb market. Because children often assist with crushing, washing, and disassembling the LABs, the potential for direct exposure through Pb dust inhalation and ingestion is especially high. In a review of 98 occupational health studies, increased BLLs were discovered among workers and nearby communities across the developing world highlighting this consistent problem, particularly in industrializing countries where regulatory capacity is limited [51].

Mass Pb poisonings triggered by this industry have been reported throughout the world, ranging from Dakar, Senegal to Dong Mai, Vietnam to villages and towns all over China [52]. Nevertheless, because of the sensitive nature of pollution research and monitoring in China, as well as the recent burgeoning of small-scale and backyard LAB enterprises, aggregate data explicitly linking these incidents to industrial Pb sources remain scarce.

### Policy responses

On November 21, 2008 the Ministry of Environmental Protection (MEP) formally promulgated “Clean Production Standards for the Lead Battery Industry” (HJ 447–2008) and implemented these in February 2009. These standards mandated application of currently available industrial technology and equipment for the LAB industry. Restrictions were also placed on natural resource usage and pollution emissions.

In response to recent mass Pb poisonings, in March 2011 the MEP along with the National Development and Reform Commission jointly issued an environmental protection special action decree (UNCED [2011] No. 41), with remediation of the LAB industry as its primary goal [44]. The central government tasked all local environmental protection bureaus to immediately conduct a thorough investigation and remediation of all environmental law violators. All LAB enterprises under investigation would be forced to comply with inspections.

As a result of this decree, by July 31, 2011 the investigation of all 1,930 known LAB enterprises (manufacturing, assembly, and recycling) resulted in the complete shutdown of 583 and discontinuation of 405 LAB enterprises. The success in quickly enforcing this special action decree was still riddled with uncertainty, as the MEP acknowledged the numerous problems with regulating smaller-scale unregistered LAB enterprises. Nevertheless, this “storm” of regulatory enforcement reflects the government’s acknowledgement of the severity of Pb pollution and its primary sources.

Also underscoring the hazards of the LAB industry is the State Council’s emphasis on controlling and regulating heavy metal pollutants in its 12th Five-Year Plan to Combat Heavy-Metal Pollution. The Plan singles out the LAB industry as a priority target and calls it “a serious threat to the health of the masses, causing widespread concern for the whole society [53].” Despite this renewed emphasis on tightening regulations on the LAB industry, the economic demand for and increased utility of LABs will contribute to further toxic emissions.

Numerous policy measures can be implemented to reduce the risk of overexposure. Currently, LAB facilities must be located at a minimum distance away from certain residences (Health Protection Distance Standard for Lead Battery Plants (GB 11659–89)) - usually 500 meters. This distance should be standardized to apply to all communities and extended to establish a safe distance from pollution sources, at least 1,000 meters for residences with children. Second, more rigorous enforcement of MEP policies regulating the LAB industry is needed, including minimum production and output standards that essentially ban establishment of small-scale LAB enterprises. Third, consolidation of the LAB industry should be mandated and expedited, as opposed to “gradually moving” existing production enterprises into industrial park zones “where conditions permit” (LAB Industry Access Conditions directive) [54]. This would limit the haphazard distribution of Pb pollution, create a more centralized and efficient industry, and streamline regulatory enforcement. Finally, requiring all LAB enterprises to install advanced air filtration, scrubbing, and water treatment technologies would reduce overall Pb emission and exposure rates.

Because of the diverse sources of Pb exposure in China, there is no one-size-fits-all policy to prevent all Pb poisoning incidents. Pb in residential paint is an important source of exposure. Despite China’s strict residential Pb paint regulations, over 50% of China’s paint has levels exceeding international norms [55,56]. In addition, recent high-profile cases revealed Pb content in children’s toys (paint and vinyl) manufactured in China, highlighting another source of Pb exposure [57]. Exposure at home from lead-glazed ceramics and cooking ware used for food preparation can lead to direct ingestion of the chemical. Pb is also used in the solder that connects water pipes, and when rusted or corroded over time, the toxin can seep into residential drinking water systems. Nevertheless, strong measures targeting the LAB industry would represent a crucial first stage in combating this public health challenge.

## Conclusions

The LAB industry is responsible for the vast majority of Pb production in China. LAB production and recycling are currently the most significant sources of Pb exposure in China, with average BLLs in children living near battery factories in developing countries at four times the current level of concern established by the World Health Organization [24]. One must also keep in mind that this health standard of 100 μg/L is currently outdated. According to the U.S. Centers for Disease Control and Prevention, “no level of lead in a child’s blood can be specified as safe” [19]. With no safe threshold of exposure to Pb, the number of children in China at risk of Pb poisoning is in the tens of millions.

This review stresses the need to mitigate the health hazards driven by the rapid growth of the LAB industry. It also demonstrates that despite extensive knowledge of Pb’s health effects, it continues to cause serious public health concerns. Most studies of the LAB industry’s health impacts have focused on a single source of Pb emissions, or at most a proximate village located near a LAB enterprise. China’s LAB industry, however, is spread over many provinces. This review validates calls for a nationwide assessment of Pb exposure pathways and levels in China and for a more comprehensive investigation into the impacts of the LAB industry. Analyses of Pb demand and its key market drivers have shown that LAB production is poised to increase dramatically and will continue to do so for many years. Thus, there is a clear urgency for immediate regulatory action to prevent further poisoning of millions of children in China.

## Abbreviations

Pb: Lead; BLLs: Blood lead levels; LAB: Lead-acid battery; E-bike: Electric bike; PV: Photovoltaic; MEP: Ministry of environmental protection.

## Competing interests

The authors declare that they have no competing interests.

## Authors’ contributions

TJV was principal investigator of the project, designed the review, and drafted the original manuscript. LH gathered and integrated data and critically reviewed the paper. CRC performed research and data analyses, contributed to the text, and critically reviewed the paper. All authors read and approved the final version of this manuscript.
